# Budesonide ameliorates lung injury induced by large volume ventilation

**DOI:** 10.1186/s12890-016-0251-z

**Published:** 2016-06-04

**Authors:** Ying-Nan Ju, Kai-Jiang Yu, Guo-Nian Wang

**Affiliations:** Department of ICU, Cancer Hospital of Harbin Medical University, Harbin, 150081 China; Department of Anesthesiology, Cancer Hospital of Harbin Medical University, Pain Research Institute of Heilongjiang Academy of Medical Sciences, No. 150 Haping Rd., Nangang District, Harbin, 150081 China

**Keywords:** Budesonide, Lung injury, Mechanical ventilation

## Abstract

**Background:**

Ventilation-induced lung injury (VILI) is a health problem for patients with acute respiratory dysfunction syndrome. The aim of this study was to investigate the effectiveness of budesonide in treating VILI.

**Methods:**

Twenty-four rats were randomized to three groups: a ventilation group, ventilation/budesonide group, and sham group were ventilated with 30 ml/kg tidal volume or only anesthesia for 4 hor saline or budesonide airway instillation immediately after ventilation. The PaO_2_/FiO_2_and wet-to-dry weight ratios, protein concentration, neutrophil count, and neutrophil elastase levels in bronchoalveolar lavage fluid (BALF) and the levels of inflammation-related factors were examined. Histological evaluation of and apoptosis measurement inthe lung were conducted.

**Results:**

Compared with that in the ventilation group, the PaO_2_/FiO_2_ ratio was significantly increased by treatment with budesonide. The lung wet-to-dry weight ratio, total protein, neutrophil elastase level, and neutrophilcount in BALF were decreased in the budesonide group. The BALF and plasma tumor necrosis factor (TNF)-α, interleukin (IL)-1β, IL-6, intercellular adhesion molecule (ICAM)-1, and macrophage inflammatory protein (MIP)-2 levels were decreased, whereas the IL-10 level was increased in the budesonide group. The phosphorylated nuclear factor (NF)-kBlevels in lung tissue were inhibited by budesonide. The histological changes in the lung and apoptosis were reduced by budesonide treatment. Bax, caspase-3, and cleaved caspase-3 were down-regulated, and Bcl-2 was up-regulated by budesonide.

**Conclusions:**

Budesonide ameliorated lung injury induced by large volume ventilation, likely by improving epithelial permeability, decreasing edema, inhibiting local and systemic inflammation, and reducing apoptosis in VILI.

## Background

Mechanical ventilation (MV) is indispensable for patients with acute respiratory distress syndrome (ARDS), and it is required for about 39 % patients in intensive care units [1]. However, MV can damage injured lungs in patients with ARDS [2]. Studies have shown that about 24 % of ARDS patients treated with MV developed ventilator-induced lung injury (VILI) [3], which resulted in a 40–50 % mortality rate [4]. MV with a large volume may lead to alveolar overstretching, increase alveolar-capillary permeability, and cause pulmonary edema [5] and lung focal inflammation [6]. Small tidal volume MV can reduce the lung injury and lower the mortality of ARDS [4]; however, ARDS remains a major problem still associated witha mortality of 25–45 % in intensive care units [7]. Therefore, it is imperative to develop alternative therapies to attenuate VILI.

Studies have shown that the imbalance of pro- and anti-inflammatory cytokines plays a critical role in the pathogenesis of VILI [8, 9]. During VILI, cytokines are released, leucocytes are recruited to the lung, and lung permeability is increased, resulting in lung edema and deterioration of pulmonary gas exchange [10]. Moreover, the cytokines released from injured endothelial and epithelial can enter the blood and cause systemic inflammation and injury to other organs.

Glucocorticoids can ameliorate the VILI [11, 12]. However, the systemic use of glucocorticoids may cause immunosuppression and steroid resistance [13]. In addition, systemic use of glucocorticoids was not found toimprove the outcome of ARDS, butinstead led to neuromuscular weakness and increased mortality risk for patients with ARDS [14]. In contrast, administration of glucocorticoids through inhalation relieves symptoms with less clinical side effects. We also found that budesonide can ameliorate the lung injury induced by one-lung ventilation or endotoxin in our clinical work and experiments [15, 16]. Other studies also have shown that budesonide can attenuate lung injury induced by chlorine gas, surfactant-depletion, or aspiration [17–20]. Therefore, we hypothesized that budesonide can reduce the incidence of VILI. In this study, we investigated the effect of budesonide on VILI using a rat model. Our data indicated that budesonide may reduce VILI, providing an alternative approach to attenuating VILI.

## Methods

### Animal experiment

All Wistar male rats were fasted and provided with water ad libitum for 24 h before the study. Twenty-four rats were randomized to three groups: a sham group (S), a ventilation group (V), and a ventilation/budesonide group (VB) (*n* = 8 per group). Rats in the V and VB groups were ventilated for 4 h with tidal volume 30 ml/kg [21, 22] (respiratory rate: 50/min, inspiratory expiratory ratio: 1:1). All rats were anesthetized using 3 % pentobarbital sodium (30 mg/kg intraperitoneally). The S group only received anesthesia. A tracheotomy was performed for rats in the V and VB groups. The caudal vein and artery were cannulated to collect blood, analyze blood gas, and perform injection. After injection of rocuronium (0.6 mg/kg), the rats in the V and VB groups received saline or budesonide 1 mg/kg by airway instillation immediately after ventilation. All the rats were maintained under anesthesia with 3 % pentobarbital sodium (10 mg/kg) and rocuronium (0.6 mg/kg) for a 1-h interval. The arterial blood analyses were performed, and the peripheral blood samples were collected at baseline (immediately after ventilation), 1, 2, and 4 h after ventilation (T0-T3). After ventilation for 4 h, all the rats were sacrificed after anesthesia, and the lungs were collected for further analysis.

### Arterial blood gas analysis

The arterial blood gases from T0 to T3 were analyzed using a Bayer Rapidlab 348 (Bayer Diognostics, Germany). PaO_2_/FiO_2_ ratios were calculated.

### Pulmonary alveolocapillary permeability

After ventilation for 4 h, the right upper lungs were weighed and then dried at 60 °C for 48 h. The ratio of wet/dry weight (W/D) was calculated.

### Preparation of bronchoalveolar lavage fluid (BALF)

BALF was collected from the left lung by infusing chilled saline (4 °C, 15 ml/kg) containing (EDTA)-2Na and withdrawal five times. Cell differentiation was determined by staining using a cytocentrifuged spin preparation (CF-RD, Sakura, Tokyo, Japan) of the BALF. The BALF was centrifuged at 1000 g at 4 °C for 15 min. After centrifugation, the BALF were immediately stored at -80 °C. The neutrophil levels in the BALF were counted with a cell counter.

### Histopathologic analysis of lung tissue

The right lower lung was fixed with 10 % formalin, embedded in paraffin, and cut into 4-μm sections. The sections were stained with hematoxylin and eosin. Two independent pathologists analyzed and scored the lung injury under light microscopy from 0 to 4 (0, minimum damage; 1, mild damage; 2, moderate damage; 3, severe damage; and 4, maximum damage), according to the assessment of alveolar congestion, edema, neutrophil infiltration in the airspace or vessel wall, hemorrhage, the thickness of the alveolar wall, and hyaline membrane formation.

### TUNEL staining of lung sections

A lobe of each right lung was examined for apoptosis using TUNEL staining with an Apoptosis Assay kit (Roche Diagnostics GmbH, Science, Mannheim, Germany). The slides were incubated with proteinase Kfor 30 min and rinsed twice with phosphate-buffered saline (PBS). Then they were immersed in TUNEL reaction mixture at 37 °C for 60 min. After washing with PBS three times, the endogenous peroxidase activity was quenched with 0.3 % H_2_O_2_ and covered with extra-avidin peroxidase, followed by immersion in adiaminobenzidine solution. The slides were counterstained with Mayer-hematoxylin, dehydrated, and mounted. The cells showing brownish staining in the nuclei were judged as apoptotic. Ten images were randomly selected from each section, and at least 1,000 cells were counted to calculate the apoptosis index by independent pathologists.

### Western blotting

The soluble protein was extracted from right lung tissue using lysis buffer containing protein inhibitors (Beyotime Biotechnology, Jiangsu, China). The concentration of the sample protein was determined using the Bradford assay. Aliquots of homogenate protein were resolved in polyacrylamide gels and transferred onto polyvinylidene fluoride membranes. The membranes were blocked with 5 % dry milk and then probed with antibodies for Bax, Bcl-2, caspase-3, phosphorylated NF-kB (Santa Cruz Biotechnology, Santa Cruz, CA, USA), and cleaved caspase-3 (Sigma-Aldrich, St. Louis, Missouri, USA), followed by incubation with horseradish peroxidase-linked secondary antibodies (Santa Cruz Biotechnology). The bands were visualized via enhanced chemiluminescence.

### Statistical analysis

All normally distributed data are presented as mean and standard deviation (SD) and were analyzed using SPSS 11.0 (SPSS, Chicago, IL, USA). The normally distributed data were analyzedusing the unpaired *t* test for a single time-point or repeated measures analysis of variance. The non-normally distributed data were analyzed using Mann-Whitney rank sum test, and histologic data were analyzed using the Wilcoxon *U*-test.

## Results

### Budesonide improves alveolocapillary permeability and the W/D weight ratio and total protein in BALF in VILI

**Fig. 1:**
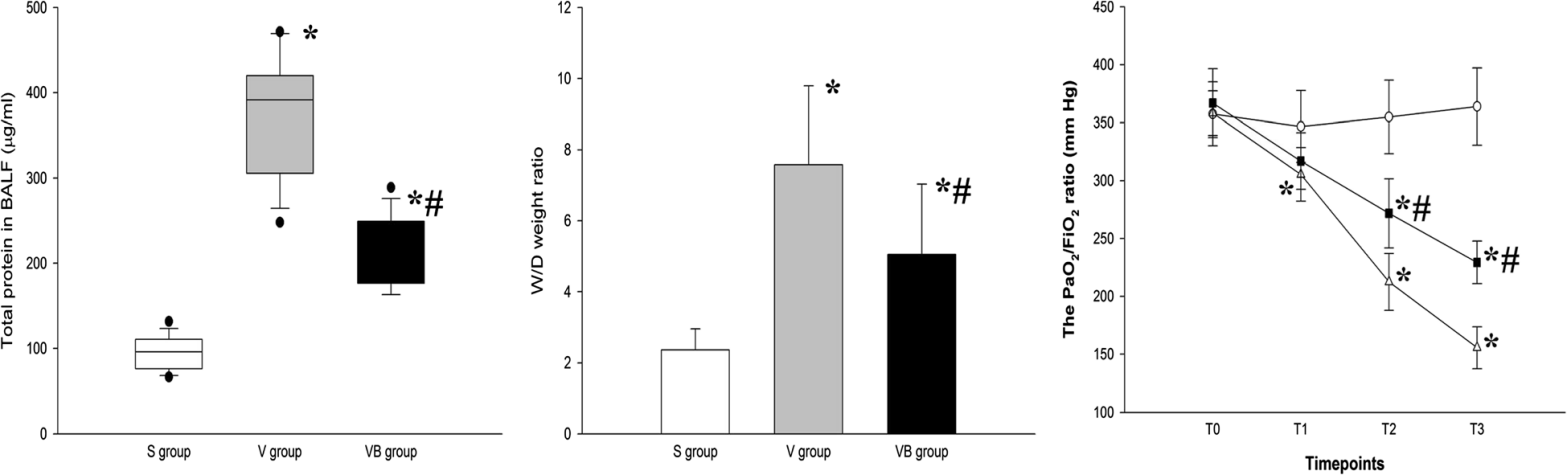
The effect of budesonide on wet/dry weight ratio, protein concentration, and PaO_2_/FiO_2_ in VILI. *P < 0.05, compared with the S group; #*P* < 0.05, compared with the V group. (, S group; , V group; , VB group)

We evaluated the effect of budesonide on alveolocapillary permeability in VILI. The results showed that the oxygen index was significantly decreased after large volume ventilation, compared with that in the S group. Budesonide dramatically increased the oxygen index in the VB group (Fig. 1). The W/D weight ratio and total protein in BALF were significantly greater in the V and VB groups, compared to the S group, but were significantly less in the VB group compared to the V group (Fig. 1). These results suggested that budesonide improved alveolocapillary permeability and the W/D weight ratio and total protein in BALF in VILI.

### Budesonide inhibits inflammation in VILI

**Fig. 2:**
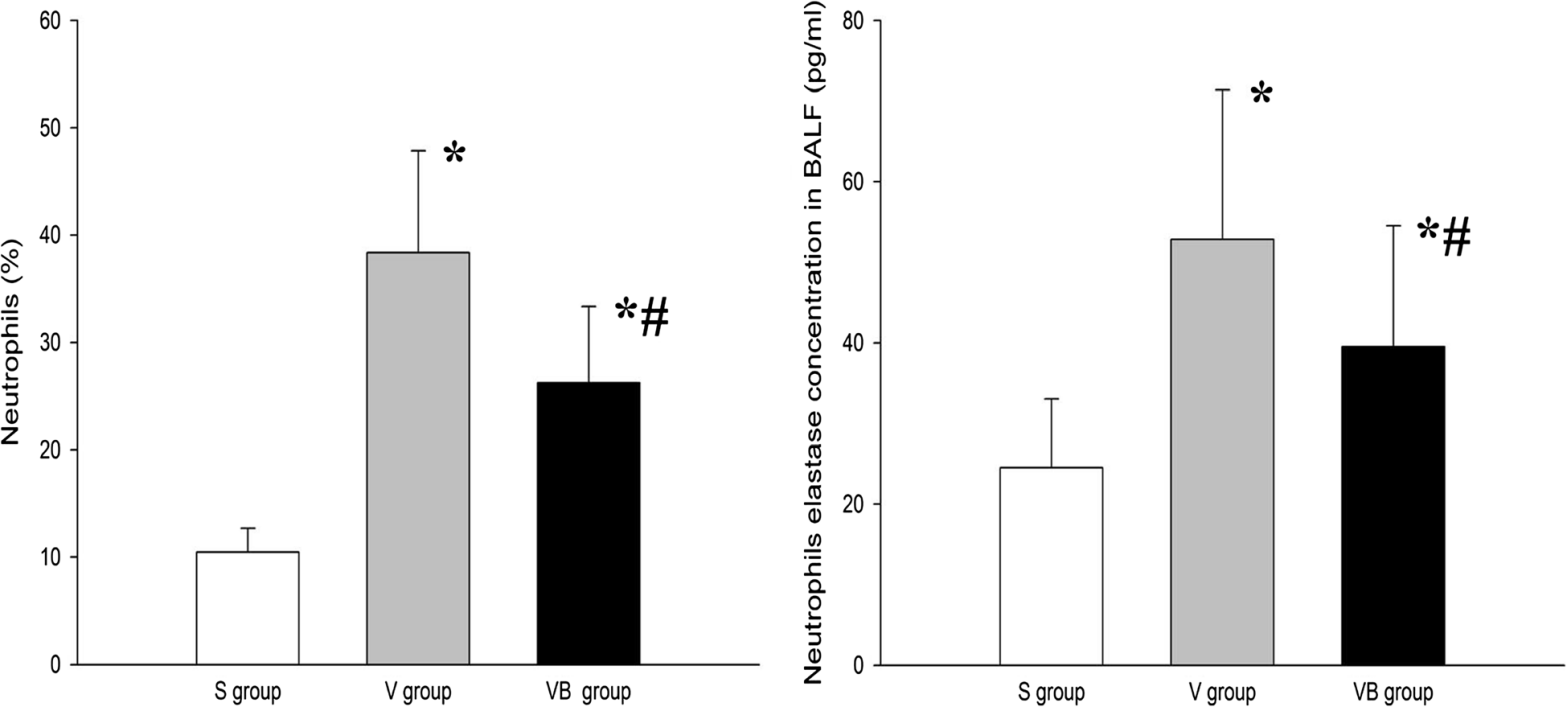
The effect of budesonide onneutrophil counts and neutrophil elastase levels in the BALF in VILI. *P < 0.05, compared with the S group; #*P* < 0.05, compared with the V group

**Fig. 3:**
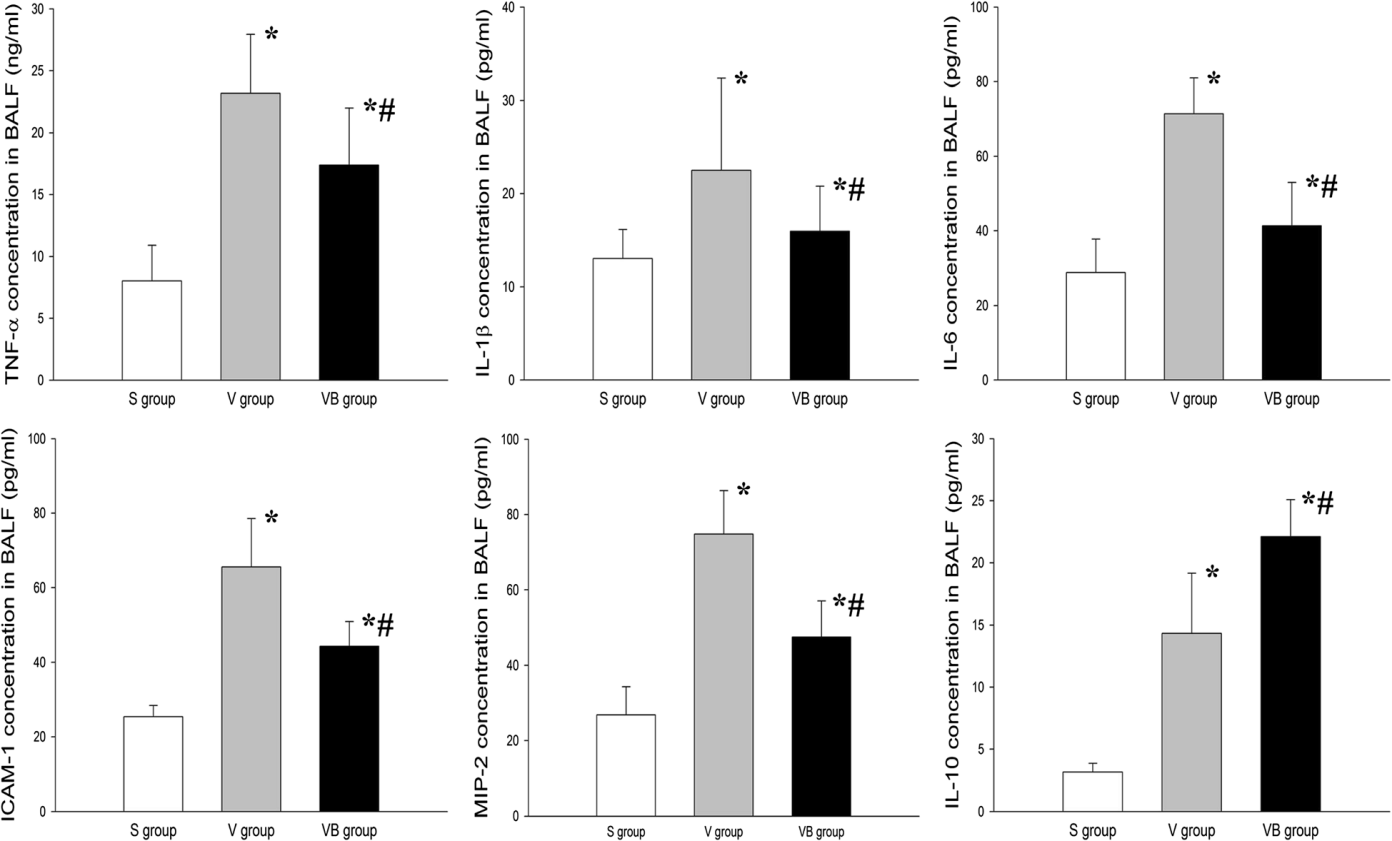
The effect of budesonide on TNF-α, IL-1β, IL-6, IL-10, ICAM-1, and MIP-2 levels in the BALF in VILI. *P < 0.05, compared with the S group; #*P* < 0.05, compared with the V group. (, S group; , V group; , VB group)

**Fig. 4:**
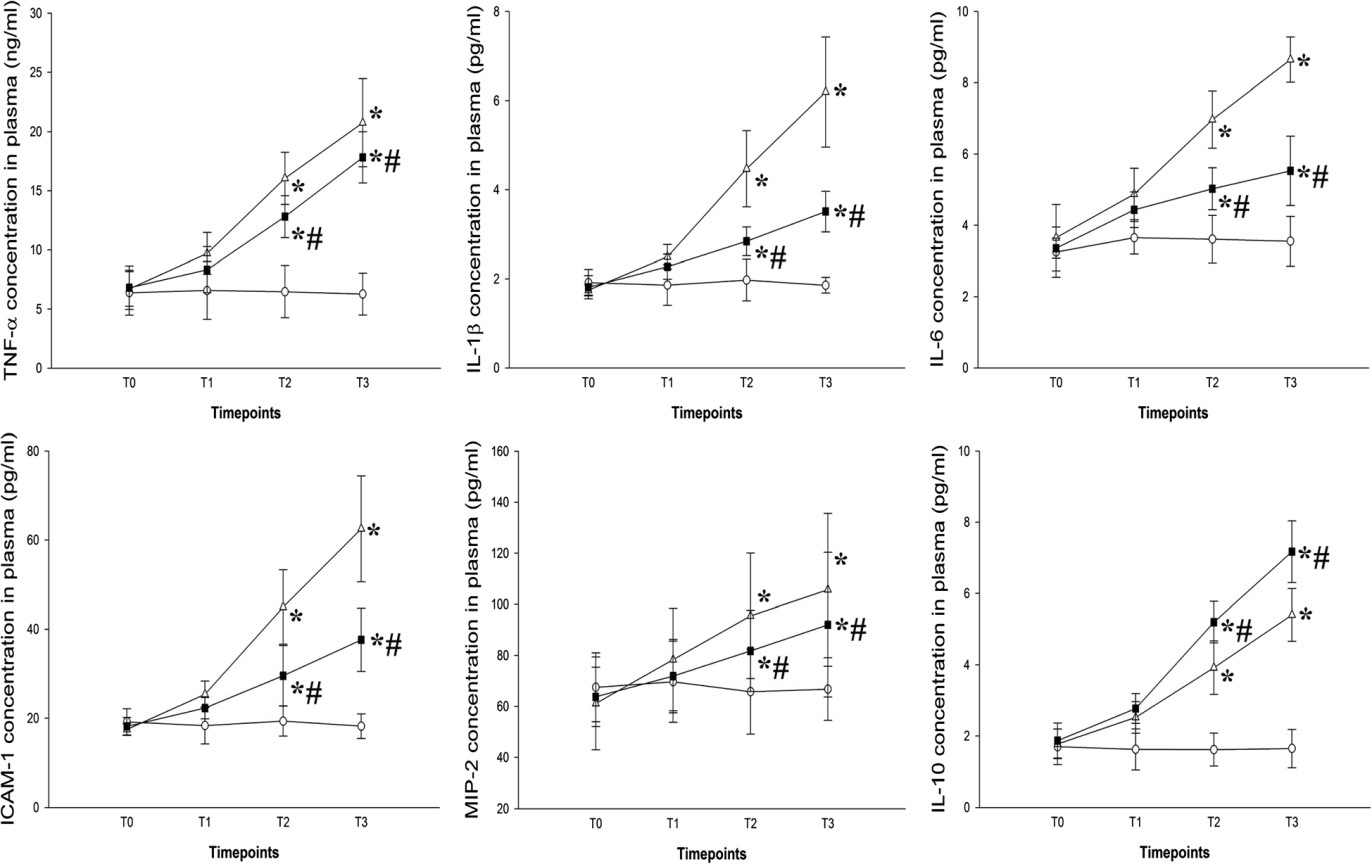
Theeffect of budesonide on TNF-α, IL-1β, IL-6, IL-10, ICAM-1, and MIP-2 levels in the plasma in VILI. *P < 0.05, compared with the S group; #*P* < 0.05, compared with the V group

**Fig. 5:**
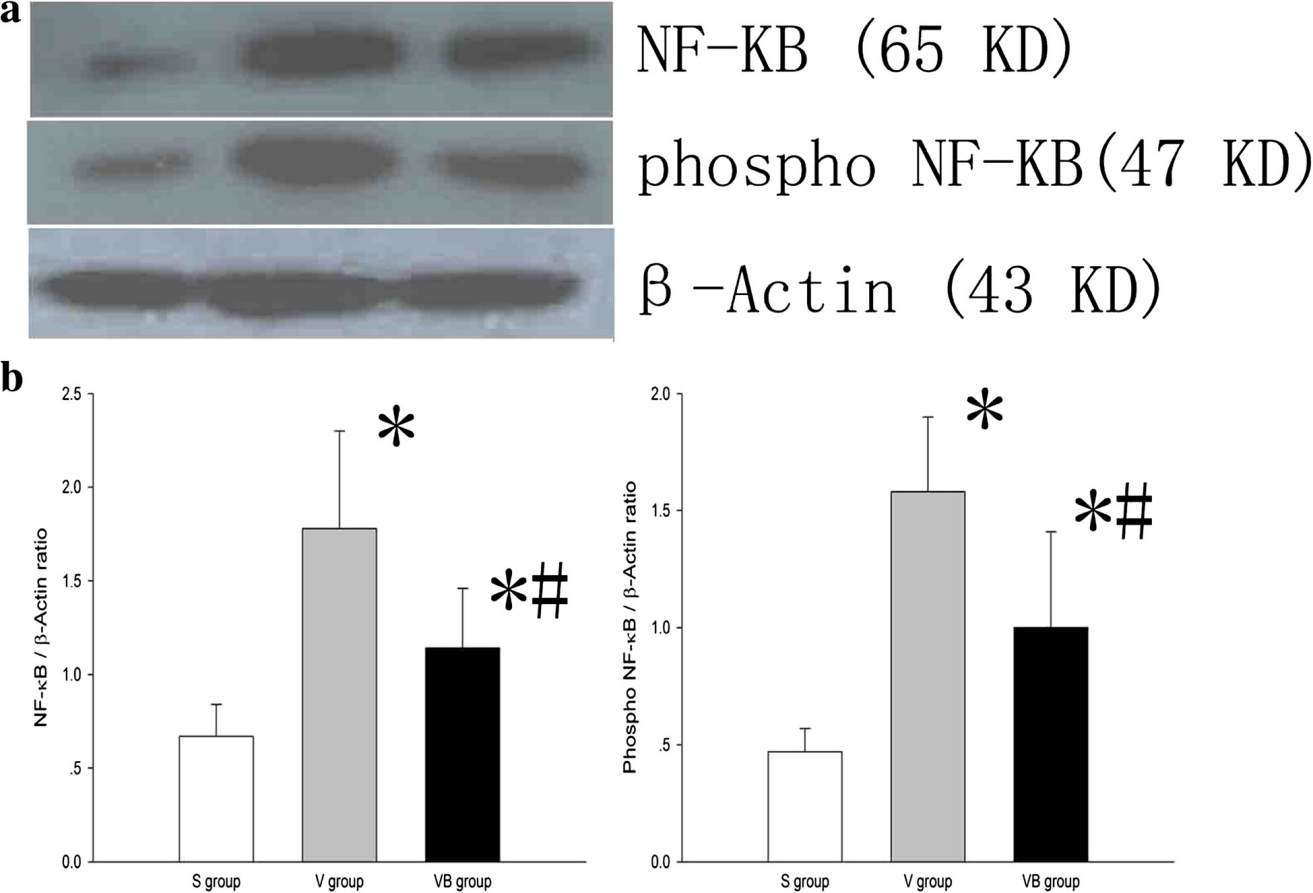
The effect of budesonide onthe expression of NF-kB and phosphorylated NF-kB in lung tissue in VILI. **a** The expression of NF-kB and phosphorylated NF-kB in 3 groups. **b** The ratio of NF-kB and phosphorylated NF-kB to β-actin in 3 groups. *P < 0.05, compared with the S group; #*P* < 0.05, compared with the V group

We evaluated the effect of budesonide on inflammationin VILI. The results showed that the levels of neutrophils in BALF were higher in the V and VB groups than inthe S group, but were significantly lower in the VB group compared to theV group (Fig. 2). In addition, the concentration of neutrophil elastase was significantly greater in the V and VB groups compared to the S group and lower in the VB group than in the V group (Fig. 2). The BALF and plasma TNF-α, IL-1β, IL-6, ICAM-1, and MIP-2 levels were significantly higher in the V and VB group than in theS group. Compared to the V group, the BALF and plasma TNF-α, IL-1β,IL-6, ICAM-1, and MIP-2 levels were significantly lower, but the IL-10 level was significantly higher in the VB group (Figs. 3 and 4). In addition, phosphorylated NF-kB was significantly up-regulated in the V and VB groups, compared with the S group. It was down-regulated by budesonide, compared with that in the VB andV groups (Fig. 5). Taken together, these data indicate that budesonide reduces local and systemic inflammation in VILI.

### Budesonide attenuates histological changes in VILI

**Fig. 6:**
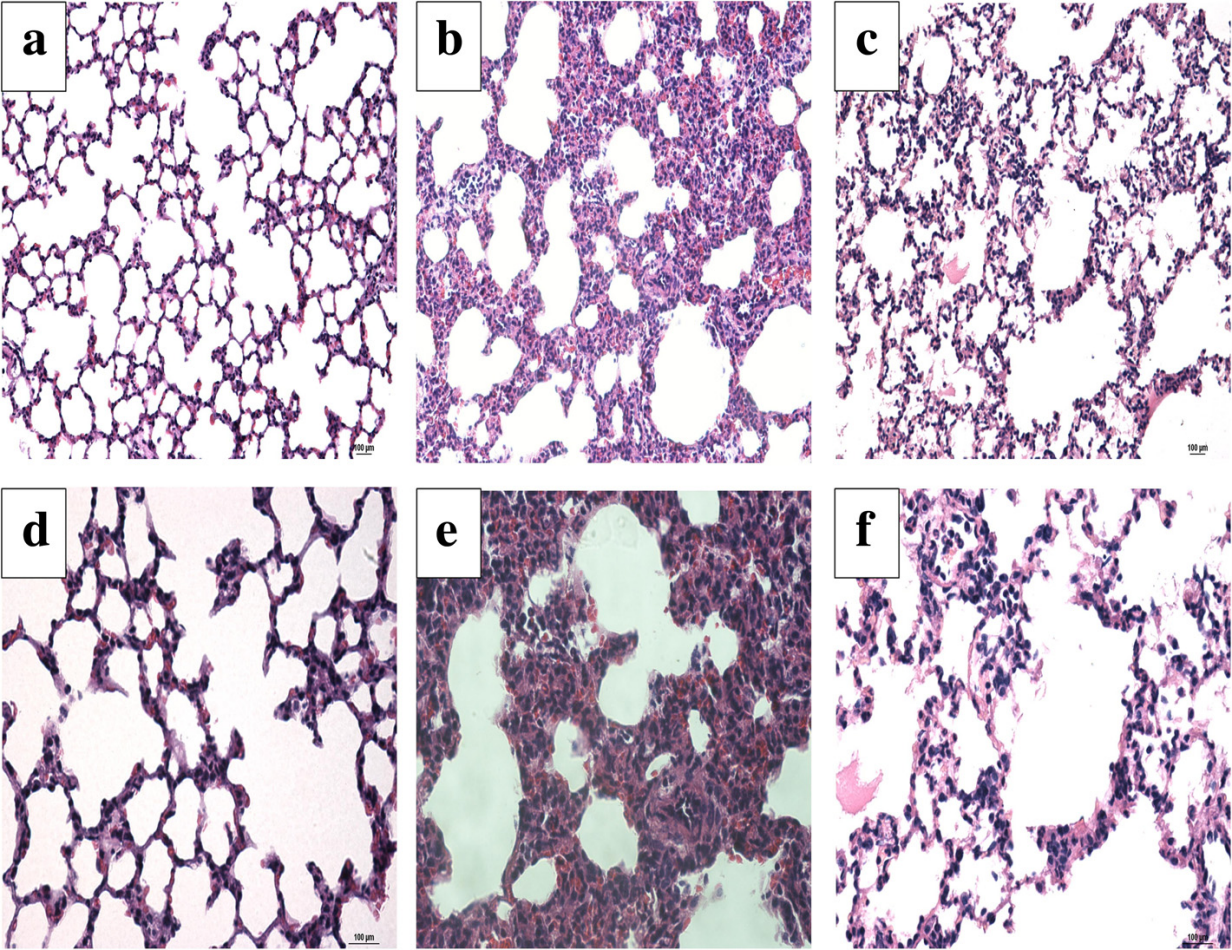
Budesonide significantly decreased the injury caused by VILI. The lung tissues were analyzed using HE staining: (**a**, **d**) S group; (**b**, **e**) V group; and (**c**, **f**) VB group. **a**-**c**, ×200; **d**-**f**, ×400

We evaluated the effect of budesonide on histological changes in VILI using hematoxylin and eosin (HE) staining. Under a light microscope, we observed typical VILI pathological changes, such as severe edema, thickening of the alveolar wall, the formation of a hyaline membrane, hemorrhage, and neutrophil infiltration in lung parenchyma in the V group, but these signs of lung tissue damage were notably reduced in the VB group (Fig. 6). The results suggest that budesonide attenuates lung injury in VILI.

### Budesonide inhibits apoptosis in VILI

**Fig. 7:**
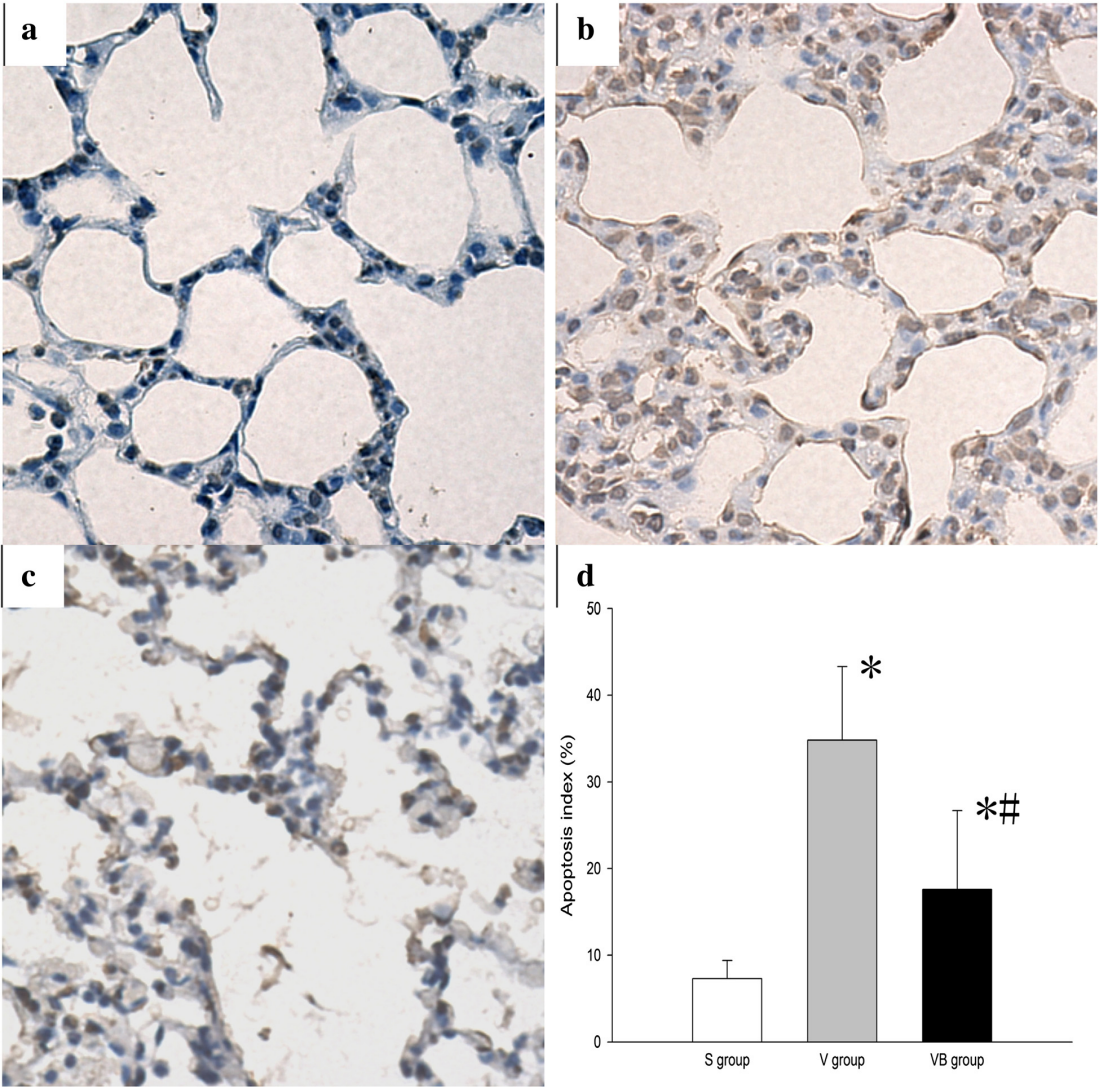
Budesonide significantly decreased VILI-induced apoptosis in lung tissues. Apoptosis among lung tissue cells was identified using TUNEL staining. Representative images of TUNEL staining of lung tissues in the S group (**a**), V group (**b**), and VB group (**c**). A-C:×400. (**d**) The apoptosis index in the three groups

**Fig. 8:**
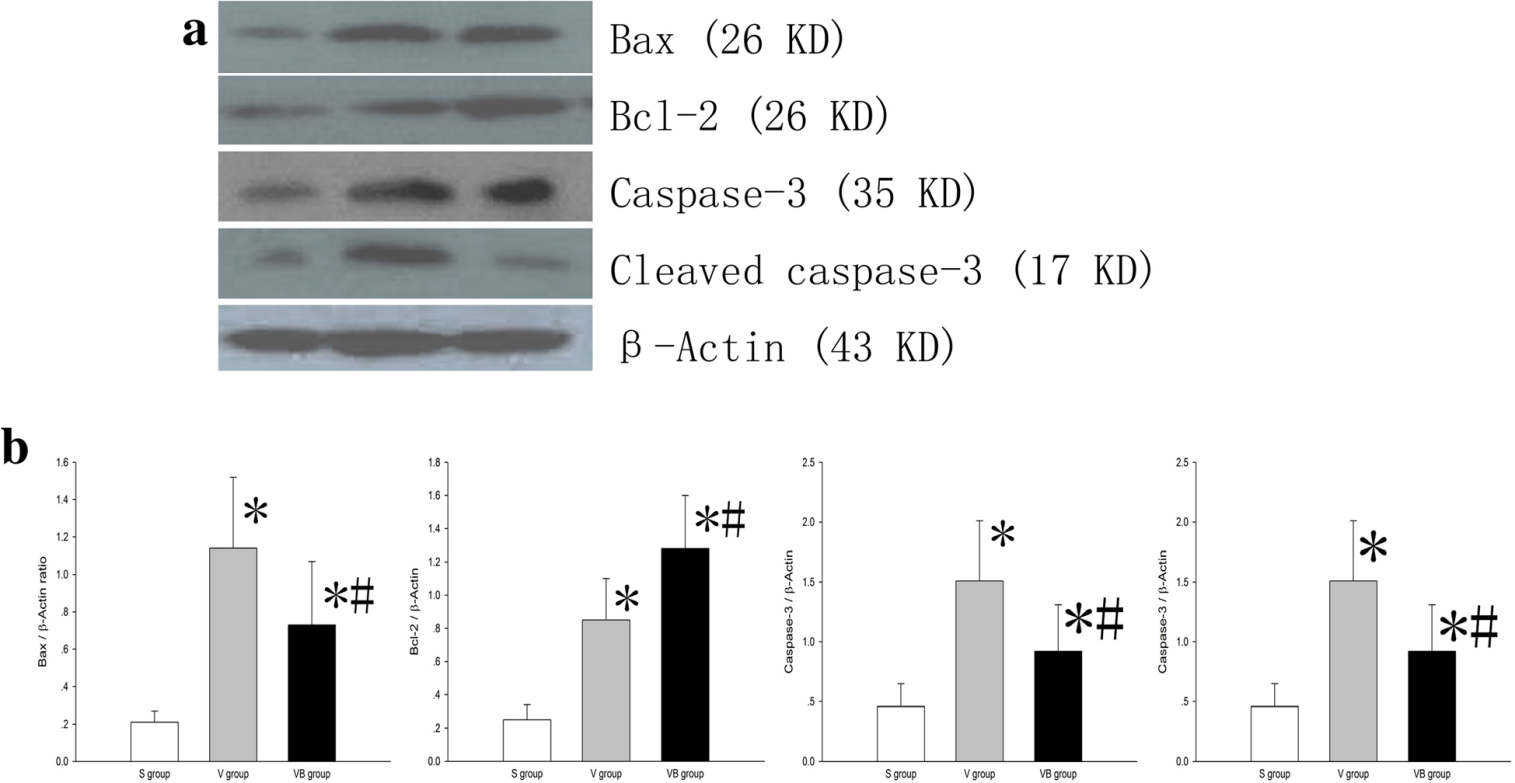
The effect of budesonide onBax, Bcl-2, caspase-3 and cleaved caspase-3 levels in VILI. **a** The Bax, Bcl-2, caspase-3 and cleaved caspase-3 levels in lung tissues were determined using Western blotting. **b** Densitometry analysis of the data shown in **a**

We evaluated the effect of budesonide on the apoptosis of lung tissues in VILI using TUNEL staining and Western blotting. We observed characteristic chromatin condensation in the nuclei of TUNEL-positive epithelial and endothelial cells in the V and VB groups, but not in the S group. These data indicated that ventilationcan induce lung cell apoptosis (Fig. 7). The number of TUNEL-positive cells was significantly decreased in the VB group, compared with the V group (Fig. 7). There were apoptotic epithelial cells, macrophages, and neutrophils in the V group tissue sections based on changes in the nuclear appearance and cell shape and position and less apoptotic epithelial cells, macrophages, and neutrophils in the VB group tissue sections (Fig. 7). The the apoptotic rates were significantly decreased in the VB group, compared with the V group (Epthelial: 32.8 % vs 17.6 %, Macrophages: 18.6 % vs 8.9 %, Neutrophils: 4.4 % vs 1.5 %). In addition, the levels of Bax, Bcl-2, caspase-3, and cleaved caspase-3 were significantly higher in the V and VB groups than in the S group. The levels of Bax, caspase-3, and cleaved caspase-3 were significantly lower and the Bcl-2 level was significantly higher in the VB group, compared to the V group (Fig. 8). Taken together, these results suggest that budesonide inhibits apoptosis in VILI.

## Discussion

MV is a life-saving treatment for patients with ARDS, but even minimal MV can induce VILI [2]. Thus, it is imperative to develop therapies that can attenuate VILI. In the current study, we found that budesonide improves alveolocapillary permeability, increases the W/D weight ratio and total protein in BALF, inhibits inflammation, attenuates histological changes, and inhibits apoptosis in VILI. Our data support that budesonide may reduce the VILI. Although several studies have indicated that budesonide or systemic glucocorticoids can reduce lung injury in various models and systemic glucocorticoids can ameliorate VILI [11, 12], this is the first study to investigate the effect of inhaled budesonide on VILI. VILI is a serious and common problem in patients who need long-term and large volume ventilation. The major injury is found in their lungs. In this study, we administered budesonide through inhalation to avoid the systemic effect of glucocorticoids and strengthen its local efficacy.

During large volume ventilation, overstretching of epithelial cells activates NF-kB and promotes NF-kB phosphorylation. Under stimulation of mechanical ventilation, many chemoattractant and proinflammatory factors, including IL-8, ICAM-1, and MIP-2, are released, and pulmonary macrophages are activated and recruit neutrophils [23, 24]. The activated macrophages and neutrophils release pro-inflammatory factors and elastase and contribute to the lung injury, leading to lung edema. In the current study, we found that budesonide improved the oxygen index, reduced histological injury in the lung, and improved lung edema after large volume ventilation. These results suggest that budesonide can protect the alveolar-capillary barrier and inhibit local inflammation. This protective effect of budesonide in VILI may be attributed to the immuno-regulation of budesonide.

In the current study, we found that budesonide significantly decreased levels of ICAM-1 and MIP-2 in VILI. ICAM-1 and MIP-2 are important adhesion molecules for neutrophils [25, 26]. In VILI, the injured epithelialand endothelial cells can release ICAM-1 and MIP-2, which recruits macrophages and causesneutrophil infiltration. The infiltrated macrophages and neutrophils further secrete proinflammatory factors and elastase, resulting in lung injury. Blockade of ICAM-1 can dramatically decrease the neutrophil influx [27] and ameliorate lung injury [28]. Therefore, budesonide likely reduced VILI by protecting the epithelial and endothelial cells from injury.

We also found that budesonide decreased the TNF-α, IL-1β, IL-6, and elastase levels and increased the IL-10 level in VILI. These results are consistent with those of previous studies [16, 29]. It has been shown that TNF-α and IL-1β are significantly elevated and play pivotal roles during the pathogenesis of VILI [30]. TNF-α,IL-1β, IL-6, and elastase are important proinflammatory factors thatnot only directly injure the lung tissue but also contribute to the aggravation of inflammation and induce cell apoptosis. IL-10 can antagonize the effect of TNF-α, IL-1β, and IL-6, and inhibit inflammatory cell migration [31]. Therefore, the budesonide-based reduction in VILI is achieved likely by regulating pro- and anti- inflammatory factors to reduce inflammation.

NF-kB is a transcription factor and a master regulator of the expression of the pro- and anti- inflammatory factors [20]. Activation of NF-kB by phosphorylation plays a pivotal role in cytokine regulation and inflammation. Inhibition of NF-kB activation can significantly decrease ALI [23, 32]. In the current study, we found that phosphorylated NF-kB were significantly up-regulated after ventilation, but dramatically down-regulated by budesonide. These data suggest that NF-kB is activated by large volume ventilation and this activation is inhibited by budesonide. Budesonide regulate the levels of pro- and anti-inflammatory cytokines probably by inhibiting activation of NF-kB.

In the current study, we also detected elevated levels of TNF-α, IL-1β, and IL-6 in the peripheral blood of rats with VILI, suggesting that the inflammation induced by VILI is not restricted to the lung and may spread to extrapulmonary organs and lead to a systemic inflammatory response and extrapulmonary organ dysfunction. This is consistent with the findings of a previous study [33]. The peripheral blood TNF-α, IL-1β, and IL-6 levels in rats with VILI were decreased after budesonide treatment. Thus, it is likely that budesonide may also reduce systemic inflammation.

Apoptosis plays a key role in VILI [22, 34]. In this study, we found apoptosis in the cells of lung tissue and this was significantly decreased by budesonide. This is consistent with previous studies that showed budesonide can inhibit apoptosis [35, 36] via inhibition of p38 MAPK phosphorylation [36]. Further, we found that the Bax, caspase-3, and cleaved caspase-3 levels were increased in VILI and the Bcl-2 level was down-regulated, but reversed to certain levels with budesonide treatment. Bax is a pro-apoptotic protein and a major regulatory checkpoint for apoptosis [37]. In contrast, Bcl-2 is an anti-apoptotic protein that can prevent activation of Bax. The ratio of Bax and Bcl-2 played a key role in the protection against or acceleration of apoptosis. Cleaved caspase-3 is the executor protein of apoptosis, will cut the DNA, and promote cell apoptosis. Both intrinsic and extrinsic pathways can activate caspase-3 and generate the cleaved caspase-3. Therefore, budesonide reduced apoptosis likely by regulating the expression of Bax and Bcl-2. In addition, we also found that macropahges and neutrophils underwent apoptosis. During VILI, the macrophages and neutrophils were activated and phagocytized the necrotic cells and then underwent apoptosis. However, in this study, we only compared the apoptosis of epithelial cells to evaluate the effect of budesonide on VILI. We can differentiate the macrophages and neutrophils from epithelial cells based on the position, shape, and nuclear characteristics of these cells.

This study has several limitations. First, rats were ventilated with a tidal volume of 30 ml/kg, which is substantially higher than volumes used in clinical application. Our preliminary study showed that a lower tidal volume (10–15 ml/kg) did not cause a decline in the PaO_2_/FiO_2_ ratio and VILI. Therefore, we increased the tidal volume to 30 ml/kg, and we successfully established the significantly decreased PaO_2_/FiO_2_and mild acute respiratory distress syndrome. Therefore, we used the tidal volume of 30 ml/kg to establish VILI. This is consistent with the study by Li et al whoalso used the 30 ml/kg tidal volume to induce ALI [21, 22]. Second, in this study, budesonide was administered at the onset of VILI, supporting the use of budesonide as a preventative treatment. Clinically, however, patients need mechanical ventilation support before dysfunction of or injury to the lung occurs. Third, we did not evaluate the purity of neutrophils in BALF, which may influence the judgment of the effects of budesonide on neutrophils in VILI. We will address these limitations in our future studies.

## Conclusion

In conclusion, in the current study, we found that budesonide ameliorated lung injury probably by improving epithelial permeability, decreasing edema, inhibiting local and systemic inflammation, and reducing apoptosis in VILI. We speculate that inhalation of budesonide reduces lung injury and edema via inhibition of NF-kB phosphorylation and decreased secretion of adhesion molecules and pro-inflammatory factors, which reduceslocal and systemic inflammation. Budesonide inhalation may be a potential approach for ARDS therapy in clinical practice.

## Abbreviations

ARDS, acute respiratory distress syndrome; BALF, bronchoalveolar lavage fluid; FiO_2_, fraction of inspired of oxygen; HE, hematoxylin and eosin; ICAM, intercellular adhesion molecule; IL, interleukin; MAPK, mitogen-activated protein kinase; MIP, macrophage inflammatory protein; MV, mechanical ventilation; NF, nuclear factor; PaO_2_, partial pressure of arterial oxygen; PBS, phosphate-buffered saline; TNF, tumor necrosis factor; TUNEL, terminal deoxynucleotidyl transferase-mediated biotinylated UTP nick end labeling; VILI, ventilation-induced lung injury; W/D, Wet/dry weight;
